# Antagonizing Retinoic Acid and FGF/MAPK Pathways Control Posterior Body Patterning in the Invertebrate Chordate *Ciona intestinalis*

**DOI:** 10.1371/journal.pone.0046193

**Published:** 2012-09-25

**Authors:** Andrea Pasini, Raoul Manenti, Ute Rothbächer, Patrick Lemaire

**Affiliations:** 1 Institut de Biologie du Développement de Marseille-Luminy (IBDML), UMR7288, CNRS/Université Aix-Marseille, Marseille, France; 2 Dipartimento di Biologia, Università degli Studi di Milano, Milan, Italy; 3 Centre de Recherche en Biochimie Macromoleculaire (CRBM), UMR5237, CNRS/Université de Montpellier, Montpellier, France; Academia Sinica, Taiwan

## Abstract

Vertebrate embryos exploit the mutual inhibition between the RA and FGF signalling pathways to coordinate the proliferative elongation of the main body axis with the progressive patterning and differentiation of its neuroectodermal and paraxial mesodermal structures. The evolutionary history of this patterning system is still poorly understood. Here, we investigate the role played by the RA and FGF/MAPK signals during the development of the tail structures in the tunicate *Ciona intestinalis*, an invertebrate chordate belonging to the sister clade of vertebrates, in which the prototypical chordate body plan is established through very derived morphogenetic processes. *Ciona* embryos are constituted of few cells and develop according to a fixed lineage; elongation of the tail occurs largely by rearrangement of postmitotic cells; mesoderm segmentation and somitogenesis are absent. We show that in the *Ciona* embryo, the antagonism of the RA and FGF/MAPK signals is required to control the anteroposterior patterning of the tail epidermis. We also demonstrate that the RA, FGF/MAPK and canonical Wnt pathways control the anteroposterior patterning of the tail peripheral nervous system, and reveal the existence of distinct subpopulations of caudal epidermal neurons with different responsiveness to the RA, FGF/MAPK and canonical Wnt signals. Our data provide the first demonstration that the use of the antagonism between the RA and FGF signals to pattern the main body axis predates the emergence of vertebrates and highlight the evolutionary plasticity of this patterning strategy, showing that in different chordates it can be used to pattern different tissues within the same homologous body region.

## Introduction

Antagonism between the Retinoic Acid (RA) and FGF signalling pathways controls the development of the posterior vertebrate body [1], [2]. The tailbud provides a posterior source of FGF8 that keeps the caudal presomitic mesoderm (PSM) cells in an immature state and maintains the proliferative status of the spinal cord precursors [1], [3]–[7]. RA produced in the segmented somites and the anteriormost PSM by the enzyme Retinaldehyde dehydrogenase2 (Raldh2) promotes PSM segmentation and triggers spinal cord patterning and differentiation [1], [8]. The signalling cascades initiated by RA and FGF inhibit each other at multiple levels. In chicken, FGF8 prevents Raldh2 and the RA receptor RARβ from being transcribed in the intermediate and posterior PSM, while RA downregulates FGF8 in the anterior PSM and the overlying neuroepithelium [1], [9]. In *Xenopus*, RA induces the expression of MKP3, a phosphatase that inhibits the MAPK branch of the FGF signalling pathway, while FGF controls the expression of Cyp26, a P450-family cytochrome that hydrolyses RA [8]. The posterior displacement of the FGF8-producing tailbud during embryo elongation results in the formation of a caudalward-travelling wave of RA signal. This determination front allows paraxial mesoderm segmentation and spinal cord differentiation to proceed coordinately with the elongation of the embryo [1], [2], [8], [10]. The opposing RA and FGF signals also control the collinear activation of Hox gene transcription: FGF activates genes located at the 5′ end of the Hox cluster, while RA induces expression of the 3′ end Hox cluster members [2], [11], [12]. Besides the RA/FGF system, a posterior-to-anterior gradient of canonical WNT activity has been described, with a role in coordinating the PSM maturation and segmentation [13]. Canonical WNT signalling also stimulates the expression of Raldh2 in the low FGF signalling environment of the anterior PSM, thus mediating the transition between FGF- and RA-sensitivity [9].

The strategy relying on opposing FGF/WNT and RA gradients to coordinate embryo elongation and patterning has been identified only in vertebrates, and it is unclear whether it is exploited by other chordates. Some support for the existence of a two opposing gradient system in the last common chordate ancestor comes from amphioxus, currently seen as the most basal extant chordate [14]. The amphioxus tailbud expresses several Wnt genes and at least one Fgf8-orthologue, Fgf8/17/18 [15], [16]; RA controls amphioxus Hox gene expression, as well as AP patterning of the spinal cord, the epidermis and epidermal neurons [17]–[19]. On the other hand, recent work only shows a role of FGF signalling in the formation of the anterior paraxial mesoderm in amphioxus, but not in the segmentation and patterning of the more posterior somites [20].

Ascidians belong to the subphylum Tunicata (or Urochordata), the sister group to vertebrates [21]. Although their larvae have a tadpole-like chordate body plan, they show a number of derived features: their embryos are made of few cells and develop according to a fixed lineage [22]; elongation of the embryo takes place by spatial rearrangement of postmitotic cells without posterior growth [23], [24]; mesoderm segmentation is absent [24]; the Hox gene cluster is disorganized and dispersed across two chromosomes; the temporal collinearity of Hox gene expression is lost and the spatial collinearity is only partially retained [25].

If the last common chordate ancestor already exploited opposing FGF/WNT and RA signals to coordinate AP elongation and patterning, then such a mechanism is likely to have undergone profound modifications in tunicates: it may have been altogether lost for lack of selective constraints, simplified, adapted to different constraints such as a determinative mode of development or recycled to fulfil novel functions. In any case, a better understanding of the molecular and functional interactions between FGF, WNT and RA pathways in the development of the posterior structures of ascidian larvae will not only contribute to clarifying the existence of a common minimum blueprint for generating the chordate body plan, but also provide insights into how the evolution of gene regulatory networks and other complex patterning mechanisms accompanies and/or underlies morphological and morphogenetic simplification in an extremely derived organism that retained a prototypical chordate body plan [22], [26], [27].

Here we show that in the elongating embryonic tail of *Ciona intestinalis*, the RA pathway is specifically active within the anterior tail epidermis, while the activities of the FGF/MAPK and WNT canonical pathways are restricted to the tailtip epidermis, a situation reminiscent of the two opposing anterior RA and posterior FGF/WNT gradients described in vertebrates. The RA and FGF/MAPK signals counteract each other, and this antagonism is required for the proper AP patterning of the tail epidermis and PNS. Thus, our data provide the first functional evidence that a strategy relying on the mutual antagonism between RA and FGF acts to pattern the posterior body of an invertebrate chordate, although in the very derived *Ciona* this may be used only to control the patterning of epidermal structures. This could reflect either a shared ancestral chordate usage of this system to control the anteroposterior patterning of all posterior body structures, followed by taxon-specific losses, or a case of repeated cooption of a particularly efficient developmental regulatory network during the course of chordate evolution.

## Results

### Regionalized activity of the RA, FGF/MAPK and canonical WNT pathways in the *Ciona* tail epidermis

To address the potential role of the RA, FGF/MAPK and WNT signals in the patterning of *Ciona* embryonic posterior structures, we first defined the territories where these pathways are active during the processes of tail patterning and elongation.

**Figure 1:**
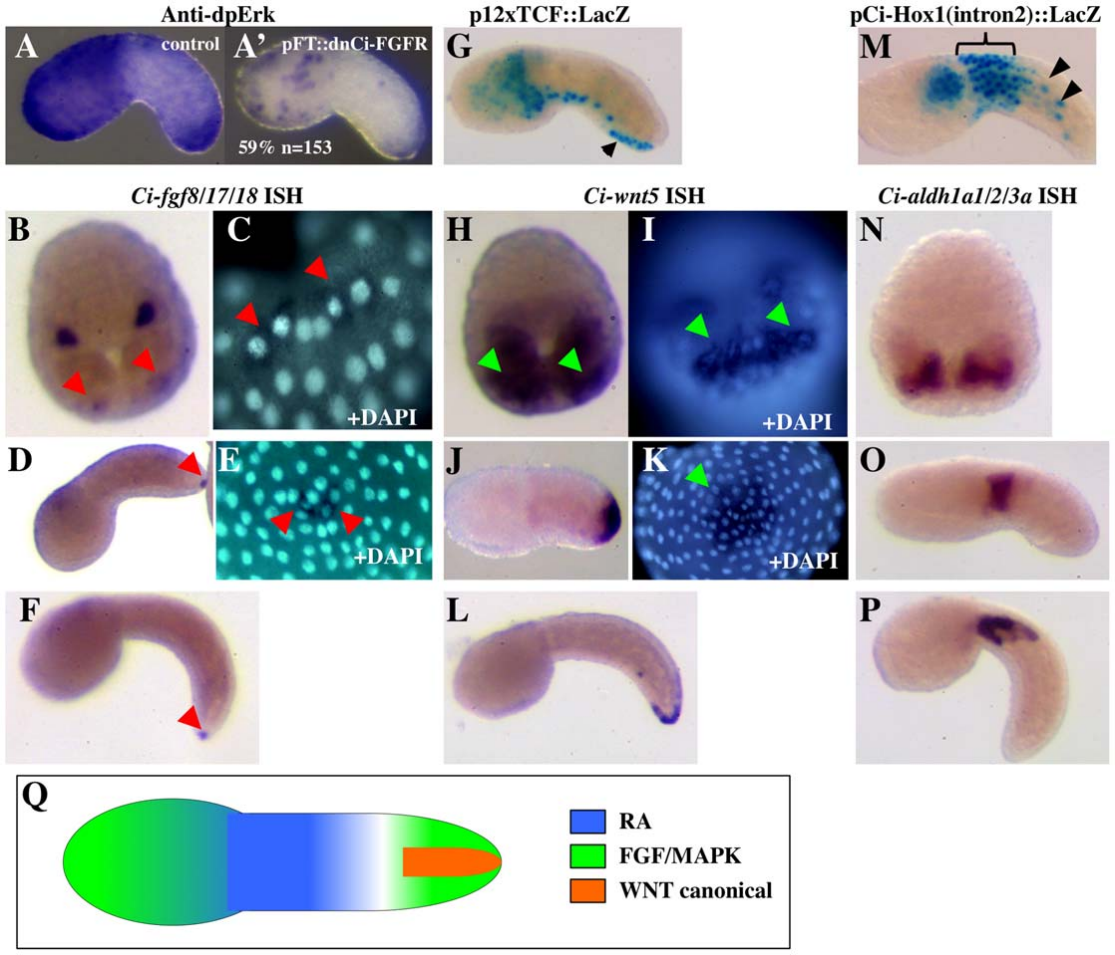
Activity of the FGF/MAPK, Wnt canonical and RA pathways in the tail epidermis of the *Ciona* embryo. (**A**): Anti dpErk immunostaining reveals MAPK pathway activity in the posterior tail epidermal cells. (**A′**): Epidermal expression of a dominant-negative form of the *Ciona* FGF receptor results in a decrease of the epidermal dpErk signal in 59% of the analysed embryos. The embryos in A and A′ are siblings and were processed in parallel. (**B–F**): *Ci-fgf8/17/18* is expressed from mid-gastrula stage onwards in a small number of epidermal cells (2–4) located at very tip of the tail (red arrowheads). (**G**): X-Gal staining of embryos electroporated with the Wnt canonical pathway reporter construct p12xTCF::LacZ shows activity in the posterior ventral midline epidermal cells (black arrowhead). (**H–L**): *Ci-wnt5* is expressed from mid-gastrula stage onwards in a group of epidermal cells located around the tip of the tail (green arrowheads). (**M**): X-Gal staining of embryos electroporated with the RA reporter construct pCi-Hox1(intron2)::LacZ shows activity in the anterior tail epidermis (bracket) and in some muscle cells (black arrowheads). (**N–P**): *Ci-aldh1a1/2/3a*, the *Ciona* homolog of the RA-synthesising enzyme Raldh2, is expressed from mid-gastrula stage onwards by the anteriormost muscle cells of the tail. **A, A′, D, E, G, J, K, M and O**: early tailbud stage embryos (stage 19–20); anterior to the left, except in E and K, posterior view. **B, C, H, I, N**: mid-gastrula stage embryos (stage 12); anterior to the top in B, H, N; posterior view in C, I. **F, L, P**: mid-tailbud stage embryos (stage 22); anterior to the left. (**Q**): Schematic ventral view of a tailbud stage embryo, recapitulating the epidermal territories where the RA (blue), FGF/MAPK (green) and Wnt canonical (orange) pathways are active.

We found that in the *Ciona* embryo tail, immunoreactivity against diphosphorylated Erk, a hallmark of the activation of the RTK-dependent MAPK pathway, is detected in the epidermal cells surrounding the tailtip from neurula [28] to tailbud stages (Fig. 1A, 2K, L). The dpErk signal is expanded by treatment with bFGF (Fig. S1A) and is suppressed or decreased following the targeted epidermal expression of a dominant-negative form of the only *Ciona* FGF receptor, *Ci-FGFR* (Fig. 1A′). Two FGF factors are expressed in the tailtip region: *Ci-fgf8/17/18*, restricted to a very small population (2 to 4 cells) of epidermal cells at the tip of the extending tail from the gastrula stage onwards (Fig. 1B–F and [29]) and *Ci-fgf9/16/20*, expressed by some tailtip muscle cells ([29] and Fig. S1B). Likewise, only two WNT genes are expressed at the end of the *Ciona* tail: *Ci-wnt5* is expressed in tailtip epidermal cells and their progenitors from the late gastrula stage and throughout tailbud stages (Fig. 1H–L); *Ci-wnt11-1/Ci-orphan wnt e* is expressed by a few tailtip muscle cells ([30] and http://aniseed-ibdm.univ-mrs.fr/gene-card.php?clusterid=cluster9820). Although WNT5 and WNT11 have often been considered as members of a “non-canonical” signalling subgroup, it has been recently shown that whether the canonical or the non-canonical pathways are activated depends more on the receptor/co-receptor context than on strictly defined WNT functional classes [31]. Interestingly, we found that *Ci-LRP5/6*, the *Ciona* homologue of LRP5/6, the WNT co-receptor required for canonical pathway activation, is expressed in the ventral midline epidermis (Fig. S2A). Consistently, WNT canonical activity, mapped with a reporter construct in which expression of β-galactosidase is under the control of multiple binding sites for the canonical WNT pathway effector, TCF [32], is restricted to two ventral territories in the *Ciona* tail, the endodermal strand and the posterior ventral midline epidermis (Fig. 1G). While the endodermal activity is the outcome of the pre-gastrula β-catenin requirement for endoderm specification [33], still visible due to the long half-life of β-galactosidase, the posterior epidermis activity likely reflects the activation of the WNT canonical pathway during tail elongation (Fig. S2B). Co-expression of an epidermally-targeted dominant-active form of β-catenin or embryo incubation with the GSK3-β inhibitor LiCl both result in ectopic reporter activity (Fig. S2C and data not shown). Finally, expression of *Ci-aldh1a1/2/3a*, the *Ciona* homologue of the RA-synthesizing enzyme Raldh2 [34], was first detected in the anterior muscle precursors at late gastrula stage, then confined to the anteriormost 3–5 muscle cell pairs throughout the process of tail extension (Fig. 1N–P and [35]). The RA-responsive territories, identified by using the reporter construct pCi-Hox1(intron2)/lacZ, in which the RA-responsive cis-regulatory sequences of the *Ci-Hox1* gene control the expression of β-galactosidase ([36] and Fig. S3A), are localized in the trunk mesenchyme and epidermis, the muscle and the anterior tail epidermis (Fig. 1M). These structures coincide with, or are adjacent to, the territories expressing *Ci-aldh1a1/2/3a*.

**Figure 2:**
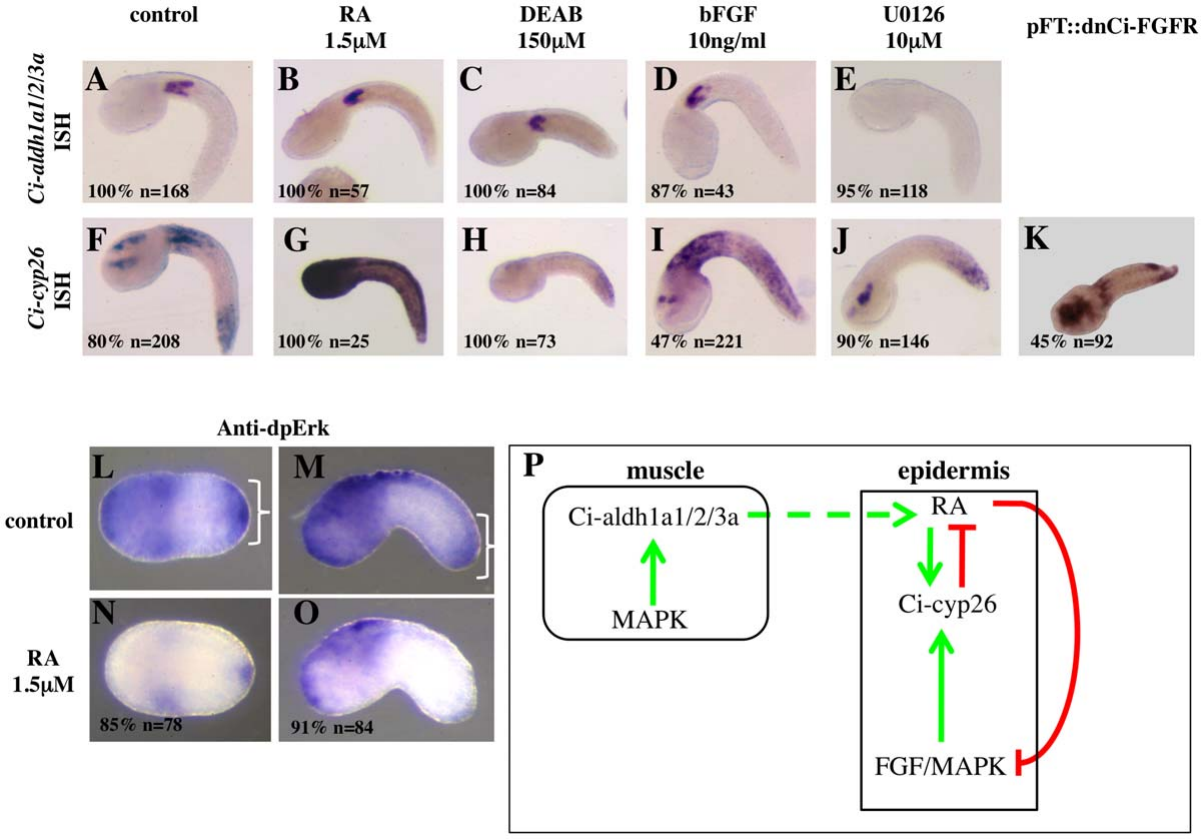
Interactions between the RA and FGF/MAPK pathways. (**A–K**): The FGF/MAPK pathway controls both synthesis and degradation of RA. The expression of the RA-synthesising enzyme *Ci-aldh1a1/2/3a* in the anterior tail muscle cells (A) is not affected by treatment with RA (B), the Ci-aldh1a1/2/3a inhibitor DEAB (C) or recombinant bFGF (D), but is suppressed by the MAPK inhibitor UO126 (E). The RA hydrolysing enzyme *Ci-cyp26* is expressed in the anterior and posterior thirds of the tail epidermis (F). RA treatment results in a strong increase of *Ci-cyp26* expression (G), while DEAB leads to its loss from the anterior tail epidermis (H). Treatment with bFGF results in the upregulation of *Ci-cyp26* expression throughout the tail epidermis (I); treatment with U0126 leads to the loss of *Ci-cyp26* expression mostly from the anterior tail epidermis (J). Epidermal expression of a dominant-negative form of the *Ciona* FGF receptor results in a decrease of the Ci-cyp26 expression (K). Treatments were performed at late gastrula stage and embryos analysed at mid-late tailbud. The percentages indicate the proportion of embryos with the phenotype shown. (**L–O**): RA inhibits transduction of the FGF/MAPK signal in the posterior tail epidermis. In control embryos, Erk diphosphorylation is detected in trunk and tailtip epidermal cells (brackets in L, M). Treatment with RA at late gastrula stage leads to a specific decrease of the dpErk signal in tailtip cells in 85% of the embryos analysed at late neurula stage (N) and 91% of embryos analysed at early tailbud stage (O). In all cases, anterior is to the left. (**P**): Diagram illustrating the interactions between the RA and the FGF/MAPK pathways in the *Ciona* tail.

In conclusion, our data reveal that, despite the major morphogenetic differences in the posterior elongation process existing between *Ciona* and the vertebrates, the regions of activity of the RA, FGF/MAPK and canonical WNT pathways in the posterior body bear striking analogies: RA is active in the anterior territories, FGF/MAPK and canonical WNT in posterior ones (Fig. 1Q).

### Reciprocal control of FGF/MAPK and RA pathways

In vertebrates, the signalling cascades triggered by RA and FGF have been found to inhibit each other at multiple levels [1], [2], [8], [9]. To address whether the RA and FGF/MAPK pathways also antagonize each other in the *Ciona* tail epidermis, we first analysed the expression of the RA-synthesising enzyme, *Ci-aldh1a1/2/3a*, and the RA-catabolising enzyme, *Ci-cyp26*, following activation or blockade of the RA and FGF/MAPK pathways between early gastrula and early tailbud stages. The expression of *Ci-aldh1a1/2/3a* is independent of RA signalling itself, as it is not modified by either exogenous RA, nor the known Raldh2 inhibitor DEAB (Diethylaminobenzaldehyde at any stage analysed (Fig. 2B, C and data not shown). As shown in Fig. 2D and data not shown, the number of muscle cells expressing *Ci-aldh1a1/2/3a* does not appear to increase in response to treatment of embryos with recombinant bFGF. On the other hand, the expression of *Ci-aldh1a1/2/3a* in the anterior tail muscle cells was lost or severely downregulated in embryos treated with the MAPK inhibitor U0126 between early gastrula and neurula stages (Fig. 2E and data not shown).

*Ci-cyp26*, the *Ciona* homologue of the cytochrome enzyme responsible for degrading RA, has a complex pattern of expression in the tail epidermis, being strongly expressed in the anterior third of the tail, more weakly in the posterior third, and absent from the central third (Fig. 2F, and [35]). A feedback control exists between RA and *Ci-cyp26*, since its expression is strongly upregulated throughout the tail epidermis by treatment with RA at all stages analysed (Fig. 2G, data not shown and [35]), and suppressed in anterior tail region by 150 mM DEAB between early gastrula and neurula stages (Fig. 2H and data not shown). At these stages, *Ci-cyp26* expression in the tail epidermis is also expanded by treatment with bFGF, albeit to a lesser extent than by RA and mostly within the anterior and middle third of the tail (Fig. 2I and data not shown). On the other hand, treatment with U0126 results in a complete loss of the anterior expression of *Ci-cyp26* (Fig. 2J), a likely secondary effect of the suppression of *Ci-aldh1a1/2/3a* expression, as this loss is rescued in embryos treated with both U0126 and RA (Fig. S3B), while epidermally-targeted expression of a dominant-negative form of *Ciona* FGFR results in the partial loss of *Ci-cyp26* expression (Fig. 2K).

Thus, our data show that the levels of RA in the *Ciona* embryo tail are regulated by both the RA pathway itself and the FGF/MAPK pathway, with MAPK controlling the production of RA within the anterior tail muscle cells and FGF promoting its degradation in the tail epidermis.

To explore whether the FGF/MAPK control of the RA pathway is mirrored by a symmetrical control exerted by RA on the FGF/MAPK activity, we analysed the phosphorylation status of Erk in the tail epidermis of *Ciona* embryos treated with 1.5 µM RA at gastrula stage. As shown in Fig. 2L–O, RA treatment leads to a decrease of Erk diphosphorylation in the epidermis, distinct from the global extinction of the signal following treatment with the MAPK inhibitor U0126 (Fig. S1A).

To summarize, multiple levels of interaction exist between the FGF/MAPK and RA pathway (Fig. 2P).

### FGF/MAPK and RA pathways control the AP patterning of *Ciona* tail epidermis

The timing of our pharmacological treatment experiments shows that the antagonism between the RA and FGF/MAPK pathways in the *Ciona* tail epidermis is set up between the mid-gastrula and late neurula stages, before the initiation of tail elongation and concomitantly with the establishment of the epidermal AP patterning [25]. We therefore explored whether the AP patterning of *Ciona* tail epidermis might be controlled by the RA and the FGF/MAPK signals.

**Figure 3:**
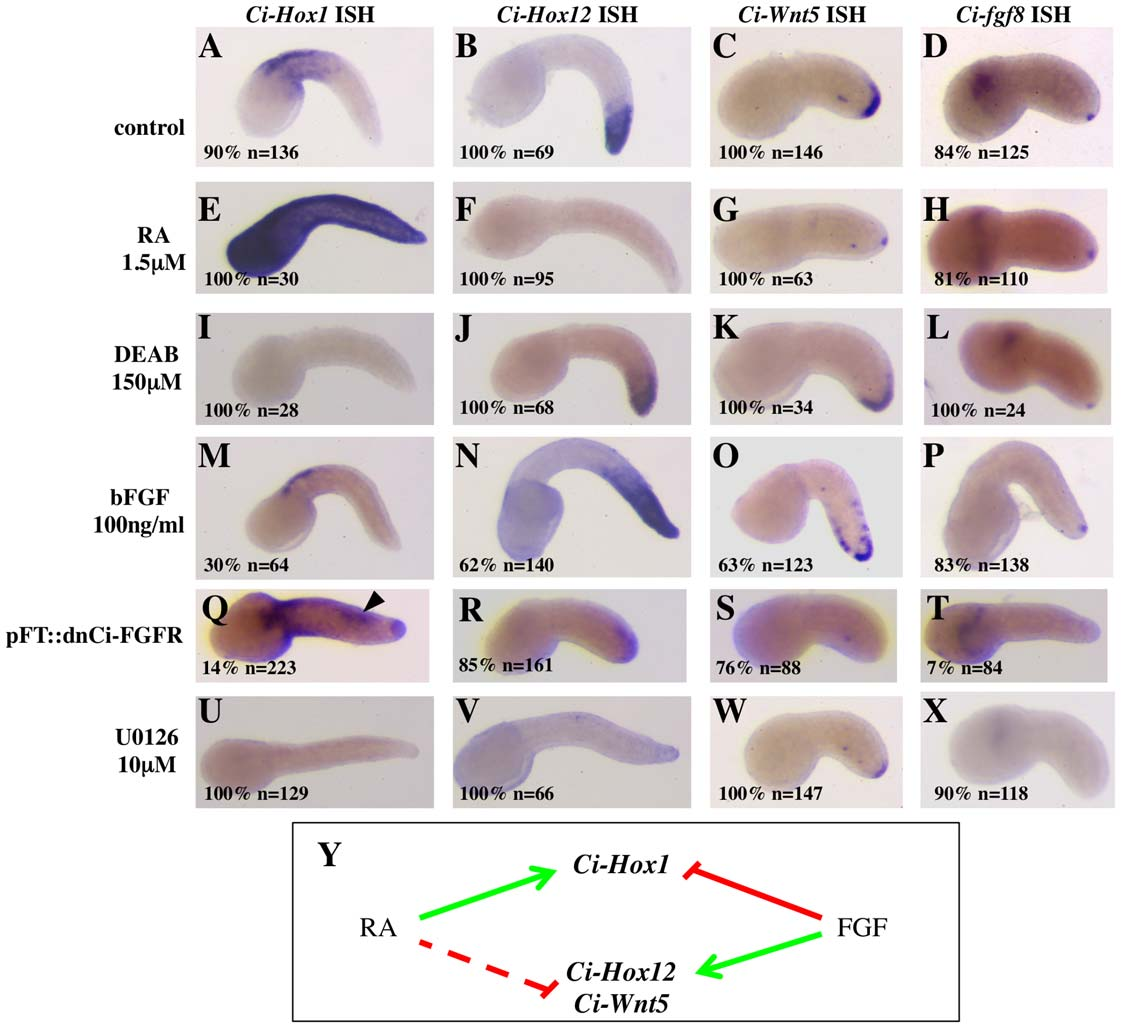
The RA and FGF/MAPK pathways control the AP patterning of *Ciona* tail epidermis. (**A–D**): In control embryos, the expression of *Ci-hox1* (A) is restricted to the anterior third tail epidermal cells, that of *Ci-hox12* (B) to the posterior third epidermal cells, while *Ci-wnt5* (C) is expressed in cells around the tailtip and *Ci-fgf8/17/18* (D) is restricted to 2–4 cells at the very tip of the tail. (**E–H**): treatment with 1.5 µM RA leads to an ectopic expression of *Ci-hox1* throughout the tail epidermis (E) and to a loss or a strong reduction of *Ci-hox12* (F) and *Ci-wnt5* (G), without affecting the expression of *Ci-fgf8/17/18* (H). (**I–L**): inhibition of the RA synthesis by treatment with 150 µM DEAB leads to a loss of *Ci-hox1* expression (I), but has no effect on *Ci-hox12* (J), *Ci-wnt5* (K) or *Ci-fgf8/17/18* (L). (**M–P**): treatment with 100 ng/ml bFGF results in a loss of *Ci-hox1* expression from the anterior tail epidermis in about 30% of the treated embryos (M). Conversely, the expression domains of *Ci-hox12* (N) and to a lesser extent of *Ci-wnt5* (O) are expanded anteriorly. The expression of *Cifgf8/17/18* is not affected (P). (**Q–T**): Epidermal expression of a dominant-negative form of the *Ciona* FGF receptor leads to the ectopic expression of *Ci-hox1* (arrowhead in Q) and to the loss or decrease of *Ci-hox12* (R), *Ci-wnt5* (S) and *Ci-fgf8/17/18* (T). The low percentage of embryos showing loss of *Ci-fgf8/17/18* expression is most likely due to the very small number of epidermal cells (2 to 4) which express *Ci-fgf8/17/18* and which fail to express dnFGFR due to the mosaicism inherent to the electroporation technique. (U-X): treatment with 10 µM U0126 results in a complete loss or a severe downregulation of both anterior and posterior genes. All embryos were treated at late gastrula stage and analysed at mid-late tailbud for *Ci-hox1* and *Ci-hox12*, at early-mid tailbud stage for *Ci-wnt5* and *Ci-fgf8/17/18*. The percentages indicate the proportion of embryos with the phenotype shown. (**Y**): Diagram showing the opposing effects of the RA and FGF signals on tail epidermis genes. Solid lines mark interactions supported by both gain-of-function and loss-of-function experiments, dotted lines those only supported by gain-of-function experiments.

The tail epidermis shows only a limited degree of AP patterning: in addition to the above described expression of *Ci-fgf8/17/18* and *Ci-wnt5* by cells at or around the tailtip, *Ci-hox12* is expressed by epidermal cells in the posteriormost third of the tail, and *Ci-hox1* by those of the anteriormost third (Fig. 3A, B and [25]). As shown in Fig. 3E–H and data not shown, treatment of embryos with 1.5 µM RA from early gastrula to neurula stages leads to an expansion of *Ci-hox1* expression throughout the entire tail epidermis and to a complete loss or a severe reduction of both *Ci-hox12* and *Ci-wnt5* expression, with only a residual signal left in cells at the very tip of the tail, while the expression of *Ci-fgf8/17/18* is not affected (Fig. 3H). When the RA signal is blocked by treatment with 150 µM DEAB at the same stages, the expression of *Ci-hox1* is completely lost from both epidermis and neural tube (Fig. 3I), whereas *Ci-hox12*, *Ci-wnt5* or *Ci-fgf8/17/18* are not significantly affected (Fig. 3J–L and data not shown).

On the other hand, treatment with recombinant bFGF from early gastrula to neurula results in an anterior expansion of the *Ci-hox12* and of the *Ci-wnt5* expression domains (Fig. 3N, O), again without affecting the expression of *Ci-fgf8/17/18* (Fig. 3P). Interestingly, bFGF treatment leads to the loss of *Ci-hox1* expression from the anterior tail epidermis, but not from the anterior neural tube, in about 30% of the embryos (Fig. 3M) and to a similar, albeit weaker, reduction of the pCi-Hox1(intron2)/lacZ reporter epidermal activity (Fig. S3C). The upregulation of *Ci-hox12* and *Ci-wnt5* following treatment with bFGF is at odds with a previous report in which SU5402, a pharmacological inhibitor of the FGF receptor, was used to rule out a role of the FGF pathway in controlling the expression of these markers [37]. To better understand the role of the FGF pathway in patterning the tail epidermis, we analysed embryos in which the reception of the FGF signal was specifically disrupted in the epidermis by means of the dominant-negative form of Ci-FGFR. As shown in Fig. 3Q–T, blocking the FGF pathway in the epidermis results in the ectopic expression of *Ci-hox1*, while leading to the decrease or loss of the expression of *Ci-hox12*, *Ci-wnt5* and *Ci-fgf8/17/18.*

Inhibition of the MAPK pathway with 10 µM U0126 leads to the complete suppression of *Ci-hox1* (Fig. 3U), revealing an action of the MAPK pathway that does not depend on the reception of the FGF signal in epidermis, and is likely related to the MAPK requirement for *Ci-aldh1a1/2/3a* expression (Fig. 2C). U0126 treatment also leads to the downregulation of *Ci-hox12*, *Ci-wnt5* and *Ci-fgf8/17/18* (Fig. 3V–X).

Overall, our data show that the RA and FGF/MAPK pathways exert partially opposing effects on the AP patterning of *Ciona* tail epidermis (Fig. 3Y): RA is required to establish and maintain the anterior identity of the tail epidermis and is sufficient to inhibit its posterior identity, while FGF is required to establish the posterior tail epidermis character, but also negatively controls the anterior epidermis identity. Therefore, the antagonism between the RA and FGF/MAPK signals in the *Ciona* tail is no mere atavism, but a functioning patterning system.

### Distinct anterior and posterior subpopulations of caudal epidermal neurons (CENs)

**Figure 4:**
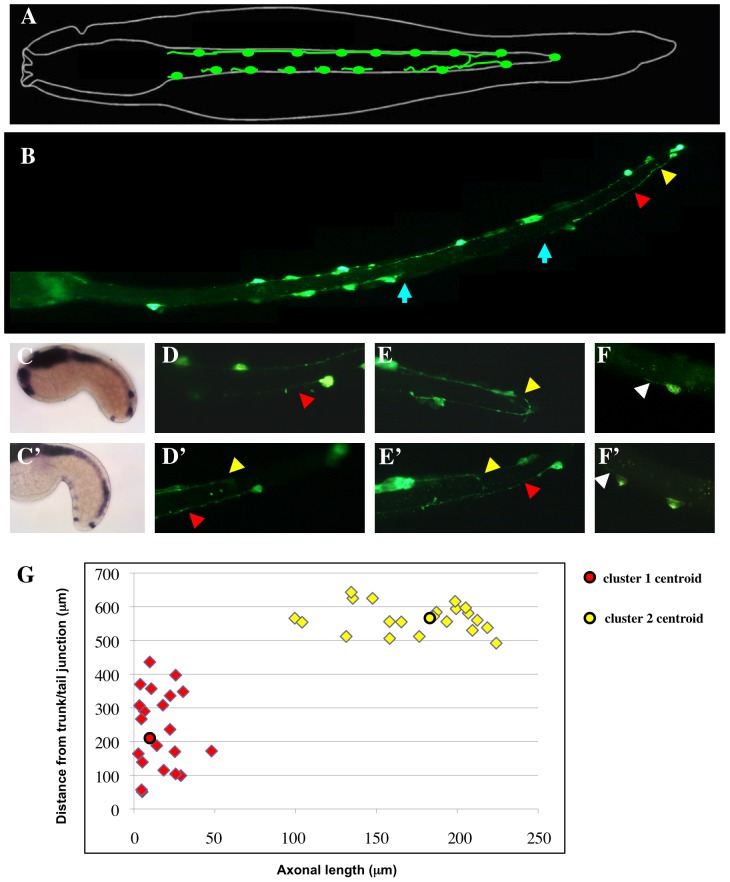
Differences between the anterior and posterior populations of *Ciona* vCENs. (**A**): Schematized representation of the *Ciona* larval CENs (adapted from [41]). (**B**): Whole mount view of a hatching larva electroporated with the pVGluT::EGFP reporter construct and immunostained with an anti-GFP antibody to show the general organization of the tail PNS. The posterior vCENs emit long axons (red arrowhead) and are contacted by axons from the dorsal CENs (yellow arrowhead), while the anterior vCENs do not establish contacts with other CENs (gap between the blue arrows). (**C, C′**): Differences in the birth time of anterior and posterior vCENs. *Ci-etr* expression in initial- (C) and early-tailbud stages (C′) embryos show that the posterior vCENs are born slightly earlier than the anterior vCENs. (**D–E′**): closeup showing the long axons of the posterior vCENs (red arrowheads) and their connexions with the dorsal CENs (yellow arrowheads). (**F, F′**): closeup showing that the anterior vCENs emit only very short axonal extensions (white arrowheads). (**G**): Quantification of the morphometric differences between anterior and posterior vCENs at hatching larva stage. The axonal length of 42 randomly chosen EGFP-expressing vCENs from 14 larvae was plotted against the position of their cell body along the tail. K-means parameter cluster analysis of the scatterplot reveals the existence of two distinct clusters of 21 points each. The centroid coordinates of the anterior (red) cluster are: axonal length = 15.9 µm, distance from trunk/tail junction = 233.8 µm, those of the posterior (yellow) cluster are: axonal length = 173,8 µm, distance from trunk/tail junction = 565.4 µm.

We next searched for differentiated *Ciona* tail structures that could be under the control of the patterning system relying on the antagonism between RA and FGF. In vertebrates, this antagonism has a role in the control of paraxial mesoderm segmentation and neuroectoderm differentiation [1], [2], [8]. Although no segmentation has been identified in *Ciona* embryos and larvae, pairs of caudal epidermal neurons (CENs) constituting the posterior larval peripheral nervous system (PNS) are distributed with some periodicity along the dorsal and ventral midlines of the tail (Fig. 4A and [38]) and, together with the acellular tail fin, are the only morphologically differentiated tail structure. We tested whether the CENs are regionalized along the AP axis by analyzing their birthdate and morphology. Examination of embryos hybridized with the neuronal-specific marker *Ci-etr* at initial- to early-tailbud stages revealed that the posteriormost ventral CENs (vCENs) are consistently born earlier than the anterior ones (Fig. 4C, C′; see also [38]). We next drove GFP expression in CENs using the *Ci-Vesicular Glutamate Transporter* (*Ci-VeGluT*) promoter [39] and analysed the morphology of the labeled CENs cell bodies and axons at the hatching larva stage. We found that all the dorsal CENs as well as the one or two posteriormost vCENs emit long axonal projections (Fig. 4B, D–E′), while the anteriormost vCENs have only short, stumpy axons or no axon at all (Fig. 4F, F′). Moreover, the posteriormost vCENs establish contact with the axonal projections of other GFP-positive cells, such as the dorsal CENs and the dorsal subepidermal bipolar cells (Fig. 4E, E′) [40], while the anterior vCENs do not appear to establish contact with any other GFP-positive cell. To quantify the morphological differences between anteriormost and posteriormost vCENs, we measured the axonal length in fluorescence microscopy pictures of 42 randomly chosen pCi-VeGluT::GFP-expressing vCENs from 14 different larvae and plotted it against the cell body position along the larval tail (Fig. 4G). K-means parameter cluster analysis of the scatterplot revealed the existence of two distinct clusters of 21 points each, whose centroid coordinates are axonal length = 15.9 µm, distance from trunk/tail junction = 233.8 µm, and axonal length = 173,8 µm, distance from trunk/tail junction = 565.4 µm respectively.

Thus, our data reveal unexpected morphological differences among *Ciona* CENs and support the existence of distinct anterior and posterior subpopulations of vCENs. Consistent with this hypothesis, posterior and anterior vCENs derive from different epidermis precursor blastomeres at the onset of gastrula and thus have a partially distinct lineage history [38]. These two neuronal subpopulations may form in response to distinct developmental programs, as it is also suggested by the observation that the anterior vCENs are absent in the distantly related ascidian *Halocynthia roretzi* [41], [42].

### FGF/MAPK, RA and canonical Wnt pathways control the number and distribution of vCENs

**Figure 5:**
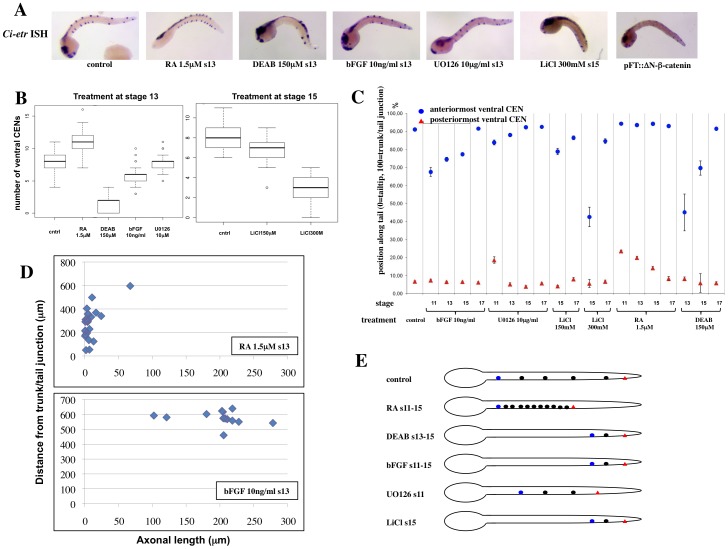
The RA, FGF/MAPK and Wnt canonical pathways control the number and position of *Ciona* vCENs. (**A**): In situ hybridization with *Ci-etr* reveals alterations in the number and distribution of the vCENs along the tail length following pharmacological or molecular interference with the RA, FGF/MAPK or Wnt canonical pathways. (**B**): Boxplot quantification of vCENs numbers (as detected by in situ hybridization against *Ci-etr*) following activation or inhibition of the RA pathway (treatment with RA and DEAB, respectively), activation or inhibition of the FGF/MAPK pathway (treatment with FGF and UO126) or LiCl-mediated activation of the Wnt canonical pathway. (**C**): Quantification of the distribution of *Ci-etr* positive vCENs along the tail length. The distance separating the anteriormost vCEN from the ventral trunk/tail junction (blue dot) and that separating the posteriormost vCEN from the tail tip (red triangle) in late tailbud embryos (stage 24) are expressed as percentage of the total tail length. (**D**): Quantification of the effect of RA or bFGF treatment on the anterior and posterior vCENs. The axonal length of EGFP-expressing vCENs from RA- or bFGF treated larvae was plotted against the position of their cell body along the tail. RA treatment (upper panel) results in the presence of the anterior, short axon vCEN population only (cluster centroid coordinates: axonal length = 8.4 µm, distance from trunk/tail junction = 276.1 µm), while bFGF treatment (lower panel) results in the presence of the posterior, long-axon vCENs population (cluster centroid coordinates: axonal length = 198.7 µm, distance from trunk/tail junction = 575.1 µm). (**E**): Schematic representation of the effect of the different pharmaceutical treatments on vCENs number and distribution.

We finally asked whether the FGF/MAPK and RA pathways, in addition to controlling the AP patterning of the tail epidermis, also affect the vCENs formation and distribution. The number and distribution of *Ci-etr*-positive vCENs at late tailbud stage (stages 23 and 24 according to [43]) were thus analyzed in embryos treated at different time points with bFGF, RA or with the pharmacological inhibitors U0126 and DEAB. In addition, embryos treated with the GSK3-β inhibitors LiCl and BIO, as well as embryos expressing a dominant-active form of β-catenin, were also analyzed to explore a possible role of the canonical Wnt pathway. As shown in Fig. 5A, B and data not shown, all the treatments tested have an effect on the number of vCENs. Treatment with RA starting at early gastrula to late neurula stages (stages 11 to 16 according to [43]) leads to a significant increase in vCEN number, mirrored by a comparable decrease in embryos treated with the RA pathway inhibitor DEAB (Fig. 5A, B and data not shown). Interestingly, posterior and anterior vCENs were differentially affected. RA stimulation leads to an increased number of anterior vCENs and to a loss of posterior ones (Fig. 5A, C). The supernumerary anterior vCENs in RA-treated embryos arise within the neurogenic midline region and are always intercalated with *Ci-etr* negative epidermal cells, thus placing RA downstream of the BMP signal which induces the neurogenic ventral midline [38] and upstream of the Delta/Notch-dependent lateral inhibition process which controls the number of CENs and epidermal cells within the midline [38]. Conversely, DEAB treatment had little effect on the posterior vCENs, but strongly affected the anterior ones (Fig. 5A, C). Consistent with the opposing activities of RA and FGF pathways during gastrula and early neurula stages, ectopic application of bFGF from the early gastrula stage repressed the formation of anterior vCENs, without effect on the posterior population (Fig. 5A, B, C). Treatment with U0126 at early gastrula stage (stage 11) led to the loss of the posteriormost CENs, and also had a weak effect on the anterior ones (Fig. 5C), likely reflecting the control of RA synthesis by MAPK. On the other hand, U0126 treatment at later stages did not affect significantly the number of vCENs (Fig. 5A, B, C). Finally, activation of the canonical Wnt pathway by treatment with either LiCl (Fig. 5A, B, C) or BIO (data not shown) or by epidermally targeted overexpression of activated β-catenin (Fig. 5A) from the mid-neurula stage represses the formation of the anterior vCENs, without affecting the posterior ones.

To correlate these observations with the morphological differences between anterior and posterior vCENs described in Fig. 4, we measured the axonal length and the cell body position along the larval tail in pCi-VeGluT::GFP-expressing vCENs from embryos treated with RA or bFGF at (Fig. 5D). In both cases, cluster analysis revealed the presence of only one cell population: in RA-treated larvae the coordinates of the cluster centroid are axonal length = 8.4 µm, distance from trunk/tail junction = 276.1 µm, while in bFGF-treated larvae they are axonal length = 198.7 µm, distance from trunk/tail junction = 575.1 µm. This shows that RA treatment results in a depletion of the posterior vCENs population, while treatment with bFGF has the opposite effect. Figure 5E summarizes the effect of the interference with the RA, FGF/MAPK and Wnt canonical signalling pathways on vCENs formation.

## Discussion

### Conservation and adaptation of a signalling and transcriptional patterning network

Although *Ciona* larvae and tailbud-stage vertebrate embryos have a remarkably similar body plan, the morphogenetic processes used to build their shared tadpole shape differ significantly. In vertebrates, embryo elongation is brought about by a combination of continuous cell proliferation in the tailbud and convergent-extension movements. On the other hand, *Ciona* embryos lack a posterior growth zone and tail elongation is mostly obtained via changes in the position and shape of postmitotic cells [23], [24]. Moreover, the AP patterning of the *Ciona* epidermis is not progressively laid down throughout the course of tail extension, but initially established between the late gastrula and early tailbud stages, then maintained during the subsequent elongation step [25]. Despite the different morphogenetic strategies exploited by *Ciona* and the vertebrates to shape their posterior body and the divergence in the differentiated structures that constitute it, we found extensive conservation of a system of opposing RA and FGF/MAPK signals to control spatial *Hox* genes expression and posterior body patterning. This is in contrast with what described in insects, where two divergent morphogenetic strategies (short- and long-germband) use different segmentation and patterning mechanisms [44], and is also surprising in view of the global divergence in orthologous gene expression patterns between ascidians and vertebrates [45].

Our data show that not only small cell-autonomous kernels based on interactions among transcription factors [27], but also a longer-range patterning mechanism relying on diffusible molecules may be conserved in its basic principles and some of its transcriptional targets over at least 500My of independent evolution. However, in the simplified context of *Ciona* embryos, the details of interaction between the RA and FGF signals differ from the model described in vertebrates. Accordingly, the mutual inhibition between RA and FGF in *Ciona* seems to take place only at the level of signal transduction or degradation rather than synthesis, as is the case in mouse or chicken [1], [2]. Just as the regulatory interactions which structure the antagonism between the two pathways differ from the vertebrate paradigm, the links between this patterning system and its final output are modified. In vertebrates, the caudal-related homeobox transcription factors of the Cdx family mediate the FGF transcriptional activation of posterior *Hox* genes [12]. In *Ciona*, the unique Cdx factor *Ci-cdx* is surprisingly expressed throughout the tail epidermis with the exception of the posteriormost cells, where FGF/MAPK signalling is active [37]. Despite these differences our data reveal an unexpected level of conservation in Hox gene regulation between *Ciona* and vertebrates: as in vertebrates, RA promotes the expression of the anterior gene *Ci-hox1* and represses the posterior *Ci-hox12*, while FGF has the opposite effect [11], [12]. Thus, although in *Ciona* the loss of temporal collinearity has allowed for (or was allowed by) the disintegration of Hox clustering, the maintenance of spatial collinearity is accompanied by the conservation of upstream regulatory factors. To what extent this holds true in other metazoans with various degrees of Hox gene clustering and temporal/spatial collinearity [46] remains an open question.

Our work thus provides a further addition to a growing list of cases where the precise relationships among regulatory network components vary from one metazoan species to the next, but the ultimate outcome of the network is comparable [44]. In particular, recent work has revealed differences in the precise interactions of the RA, FGF and Wnt signalling cascades during posterior axis elongation and segmentation in different vertebrates [47]. Network wiring differences between *Ciona* and the vertebrates may be related to the different dynamics of the antagonistic RA and FGF activities: rather than generating a moving determination front that progressively shifts posteriorly during elongation, in *Ciona* embryos RA and FGF define two stable determination fields in the epidermis before the onset of the tail extension process or at its very beginning.

### Plasticity of an ancestral chordate strategy?

In vertebrates the antagonism between RA and FGF has been shown to control the patterning of the paraxial mesoderm and spinal cord [1], [2], whereas our data show that in *Ciona* the opposition between these two signals is required to pattern the posterior epidermis. This raises the possibility that in ancestral chordates, the RA/FGF antagonistic coupling was exploited to regulate the patterning of a broad range of embryonic derivatives, including mesoderm, neurectoderm and non neural epidermis, and that in the course of evolution, different chordate taxa have undergone germ layer-specific losses of this patterning mechanism. Such a situation could reflect taxon-specific differences in the degree of patterning complexity of each germ layer, exemplified by the complex patterning of epidermis and the absence of paraxial mesoderm patterning in *Ciona*. Alternatively, it is possible that in some cases the requirement for the antagonism between RA and FGF has been overlooked. As an example, it has been shown in amphioxus that RA affects the AP patterning of the epidermis and the distribution of the epidermal neurones [18], but it is currently not known whether and how FGF also affects these processes; FGF controls the segmentation and patterning of the anteriormost Amphioxus somites [20], but the role of RA in the segmentation and patterning of the paraxial mesoderm is still unclear. Similarly, almost no data is currently available concerning the AP patterning of trunk epidermis in vertebrates and the role that the antagonism between RA and FGF signals might exert on this process.

### Morphological heterogeneity of the *Ciona* CENs: a driving force for AP tail patterning?

Our work reveals the existence of subpopulations of Ciona CENs with distinct morphologies and different susceptibility to the RA, FGF and Wnt signals. The morphological heterogeneity of the tail PNS neurons is most likely imposed by their function during the *Ciona* life cycle, which is still unknown. Whatever its function is, it is tempting to speculate that in *Ciona*, the presence of a strongly regionalized tail PNS represents the selective force requiring the maintenance of a complex signalling system responsible for controlling the AP pattern of the posterior body. In this respect, it is worth noting that in the larvacean *Oikopleura dioica*, which lacks a tail PNS analogous to that of *Ciona* (H. Nishida, personal communication), most of the RA signalling machinery is missing [48] and the Hox cluster is completely disorganized [49]. Confirmation of this hypothesis will require further investigation of the tail AP patterning, as well as of the PNS anatomy, development and function in a wider, evolutionarily significant range of ascidians and tunicates.

## Conclusions

We show that, regardless of the different morphogenetic strategies they adopt to build the chordate body plan, the vertebrates and the tunicate *Ciona intestinalis* exploit a very similar mechanism, based on the antagonism between the RA and FGF/MAPK signals, to pattern the posterior body of their embryos and larvae. Thus this mechanism, until now described only in vertebrates, likely represents an ancestral trait common to all olfactores. Remarkably, while in the vertebrates the opposition between RA and FGF/MAPK coordinates the patterning of the posterior neural tube and paraxial mesoderm, in *Ciona* it controls the AP patterning of the posterior epidermis and the associated PNS, suggesting a high degree of functional plasticity throughout evolution. We also propose that the presence of a relatively complex posterior PNS might have been the selective constraint responsible for retaining the RA vs FGF/MAPK antagonism in *Ciona*. Altogether, our work supports the existence of a common minimum blueprint for generating the chordate body plan but also shows how this has been modified and restricted or redeployed to only a subset of tissues in an extremely derived organism that retained a prototypical chordate body plan.

## Materials and Methods

### Embryo culture and pharmacological treatments

*C. intestinalis* adults were collected by the Biological Sample Collection Service of the Station Biologique de Roscoff, France. Fertilization and embryo culture were performed as previously described [50]. Embryos were treated with soluble recombinant human bFGF (10 ng/ml; Sigma), UO126 (10 µM; Calbiochem), or LiCl (150 mM or 300 mM), as described in [32], [50]. Treatment with RA (1.5 µM; Sigma) or DEAB (150 µM; Sigma), were performed according to [36].

### Constructs and electroporation

Embryo electroporation was performed as described in [38]. The following constructs were used: pCi-Hox1(intron2)::lacZ [35]; p12xTCF::LacZ [32]; pVGluT::EGFP [39], pFT::dnFGFR [51], pFT::ΔN-β-catenin [32]. The pFT plasmid contains a sequence of the *Ciona Fucosyl Transferase* control region that drives expression in the b-line from late gastrula stage (Rothbächer et al., in preparation).

### In situ hybridization, X-gal staining and immunofluorescence

In situ hybridization and X-gal staining were performed as described in [32]. Anti-EGFP staining was performed using a rabbit anti-GFP antibody (1/200, Torrey Pines Biolab), followed by an Alexa 488-conjugated anti-rabbit antibody (1/200, Molecular Probes).

### Anti-dpErk immunostaining

Detection of diphosphorylated Erk was performed with a modified version of the protocol described in [50]. Briefly, embryos were fixed for 30′ in 4% paraformaldehyde, 0.2% glutaraldehyde in artificial sea water, supplemented with 50 mM NaF and 100 mM Na_3_VO_4_ to inhibit endogenous phosphatases. After EtOH dehydration, embryos were re-hydrated in PBTri (PBS+ Triton 0.1%), treated for 10′ with H_2_O_2_ 0.6% in PBTri, blocked in PBTri, 1% Roche Blocking Reagent, 10% goat serum, then incubated overnight with an anti-dpErk antibody (1/1200, Sigma). After extensive washing in PbTri, embryos were incubated overnight with a HRP-conjugated anti-mouse antibody (1/200, Molecular Probes), followed by revelation with the TSA Plus Fluorescein Kit (Perkin Elmer). After the revelation, the embryos were fixed for 30′ in 4% paraformaldehyde, extensively washed in PBTri, then blocked and incubated overnight with an AP-conjugated anti-fluorescein antibody (Roche), followed by NBT/BCIP staining.

### Quantification of vCENs number, position and morphology

vCENs were stained by ISH against Ci-etr, and their number was counted in control and treated embryos. To quantify their distribution along the tail, embryos were photographed and analysed with the ImageJ software. The following parameters were analysed: total embryo length (from palps to tailtip); total tail length (from the ventral junction between trunk and tail to the tail tip); anteriormost vCEN position (distance between the trunk/tail junction and the anteriormost Ci-etr+ cell); posteriormost vCEN position (distance between the tailtip and the posteriormost Ci-etr+ cell). All the statistical analyses on neuron number and position were performed using ANOVA with Tukey post hoc test in R environment. To quantify the morphological differences between anterior and posterior vCENs, fertilized eggs were electroporated with the pVGluT::EGFP construct, then let develop until hatching larva stage in controls condition or in the presence of RA or bFGF from late gastrula stage. The larvae were processed for anti-GFP immunofluorescence as described above, then individual vCENs were photographed and analysed with the ImageJ software. The following parameters were measured: cell body surface, axonal length and distance of the cell body from the trunk-tail junction. Cluster analysis was performed with the TANAGRA software (http://chirouble.univ-lyon2.fr/~ricco/tanagra/en/tanagra.html).

## Supporting Information

Figure S1 (**A**): treatment with recombinant bFGF leads to the ectopic diphosphorylation of Erk, while treatment with U0126 results in a loss of the dpErk signal throughout the embryo, distinct from the tailtip specific loss due to RA treatment shown in Fig. 2L–O. (**B**): *Ci-fgf9/16/20* is expressed by a few muscle posterior muscle cells close to the tail tip (image kindly provided by C. Hudson and H. Yasuo).
(TIF)

Figure S2 (**A**): *Ci-LRP5/6*, the *Ciona* homologue of LRP5/6, the WNT co-receptor required for canonical pathway activation, is expressed in the ventral midline epidermis. Left panel, side view of an early tailbud stage embryo, anterior is to the left. Right panel, ventral view of the same embryo, anterior is to the top. (**B**): X-Gal staining of embryos electroporated with low amounts of the Wnt canonical pathway reporter construct p12xTCF::LacZ. Following mosaic inheritance of the electroporated plasmid, the activity is detected in the posterior ventral midline tail epidermis, but not in the endodermal strand. The epidermal activity is independent from the endodermal activity and is first detected at neurula stage in the precursors of the posterior ventral midline epidermis. (**C**): Co-expression of an epidermally-targeted dominant-active form of β-catenin leads to ectopic canonical Wnt activity. Black arrows point to the posterior ventral tail midline.
(TIF)

Figure S3 (**A**): treatment with DEAB blocks the activity of the pCi-Hox1(intron2)::lacZ reporter construct, while treatment with RA leads to its ectopic activation throughout the embryo. (**B**): treatment with RA at late gastrula stage rescues the U0126-induced loss of Ci-cyp26 expression in the anterior tail epidermis. (**C**): bFGF treatment of pCi-Hox1(intron2)::lacZ electroporated embryos results in a decrease in the number of embryos showing epidermal activity.
(TIF)
