# Supplementary figures and images for: Antagonizing Retinoic Acid and FGF/MAPK Pathways Control Posterior Body Patterning in the Invertebrate Chordate Ciona intestinalis
Source: PLoS One. 2012 Sep 25;7(9):e46193. doi: 10.1371/journal.pone.0046193 (PMC3458022; doi:10.1371/journal.pone.0046193)

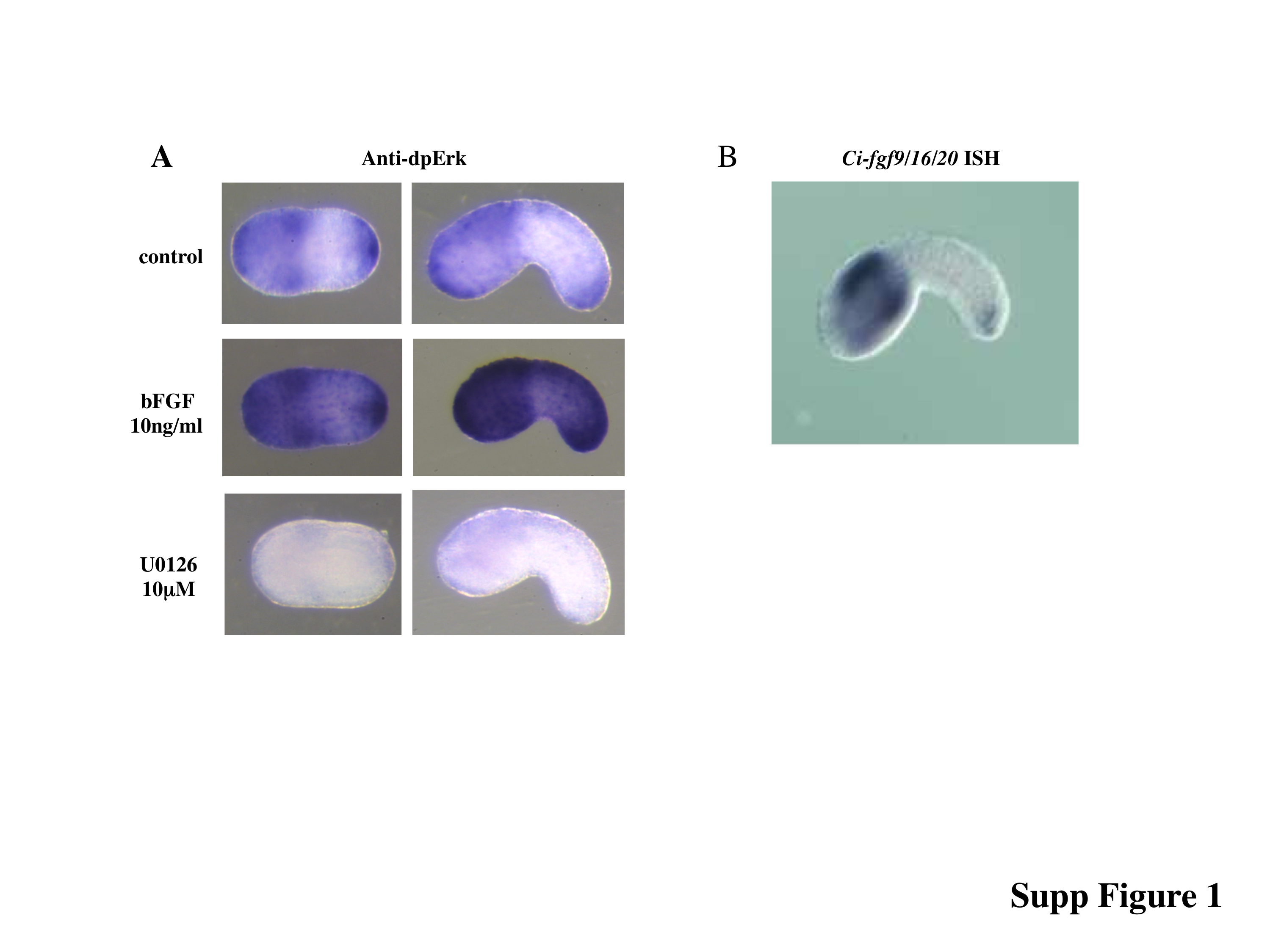

Supplement: Figure S1 — (A): treatment with recombinant bFGF leads to the ectopic diphosphorylation of Erk, while treatment with U0126 results in a loss of the dpErk signal throughout the embryo, distinct from the tailtip specific loss due to RA treatment shown in Fig. 2L–O. (B): Ci-fgf9/16/20 is expressed by a few muscle posterior muscle cells close to the tail tip (image kindly provided by C. Hudson and H. Yasuo). (TIF) [file pone.0046193.s001.tif]

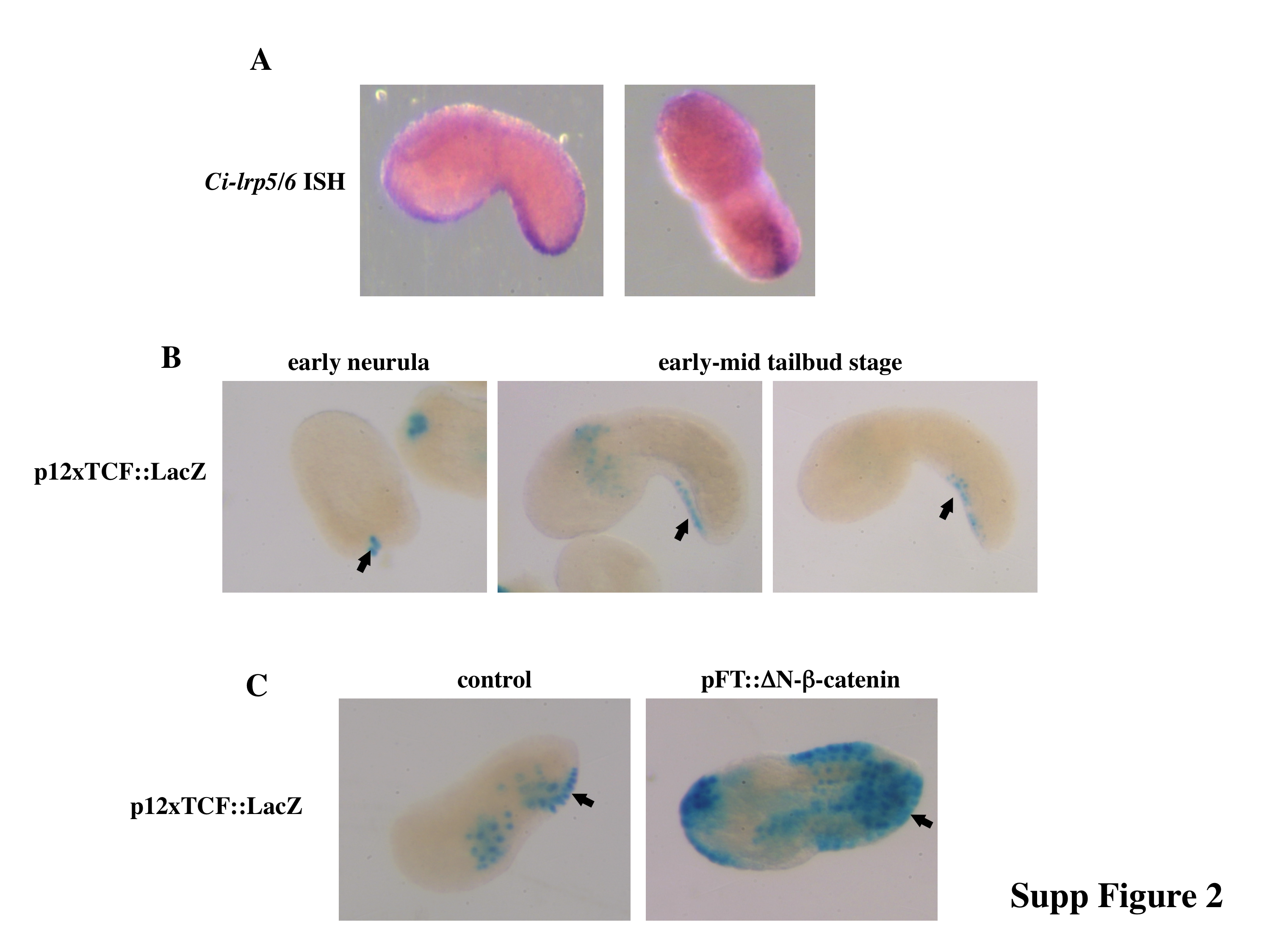

Supplement: Figure S2 — (A): Ci-LRP5/6, the Ciona homologue of LRP5/6, the WNT co-receptor required for canonical pathway activation, is expressed in the ventral midline epidermis. Left panel, side view of an early tailbud stage embryo, anterior is to the left. Right panel, ventral view of the same embryo, anterior is to the top. (B): X-Gal staining of embryos electroporated with low amounts of the Wnt canonical pathway reporter construct p12xTCF::LacZ. Following mosaic inheritance of the electroporated plasmid, the activity is detected in the posterior ventral midline tail epidermis, but not in the endodermal strand. The epidermal activity is independent from the endodermal activity and is first detected at neurula stage in the precursors of the posterior ventral midline epidermis. (C): Co-expression of an epidermally-targeted dominant-active form of β-catenin leads to ectopic canonical Wnt activity. Black arrows point to the posterior ventral tail midline. (TIF) [file pone.0046193.s002.tif]

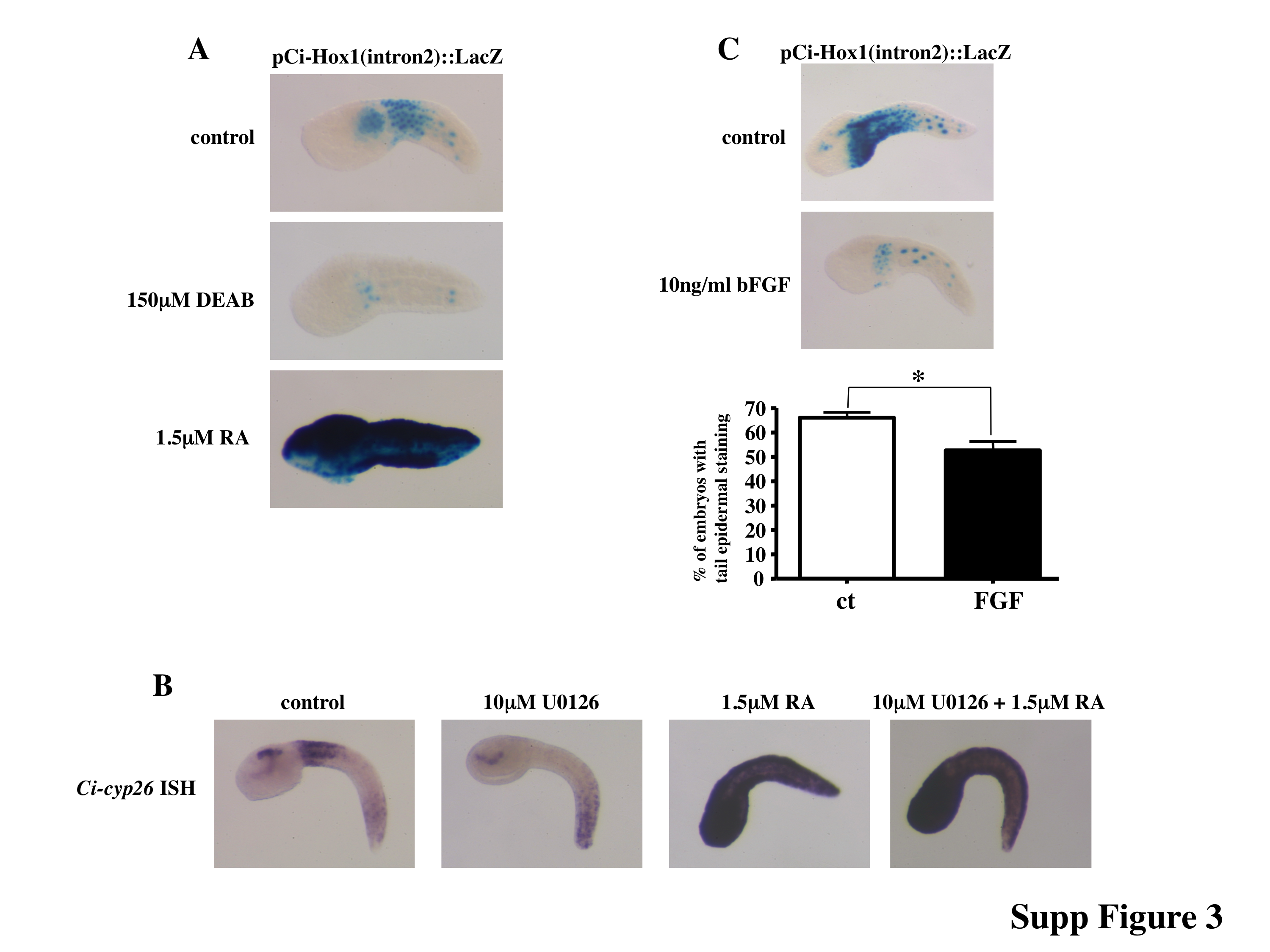

Supplement: Figure S3 — (A): treatment with DEAB blocks the activity of the pCi-Hox1(intron2)::lacZ reporter construct, while treatment with RA leads to its ectopic activation throughout the embryo. (B): treatment with RA at late gastrula stage rescues the U0126-induced loss of Ci-cyp26 expression in the anterior tail epidermis. (C): bFGF treatment of pCi-Hox1(intron2)::lacZ electroporated embryos results in a decrease in the number of embryos showing epidermal activity. (TIF) [file pone.0046193.s003.tif]
